# Oxidized mitochondrial DNA sensing by STING signaling promotes the antitumor effect of an irradiated immunogenic cancer cell vaccine

**DOI:** 10.1038/s41423-020-0456-1

**Published:** 2020-05-12

**Authors:** Chunju Fang, Fei Mo, Li Liu, Jing Du, Min Luo, Ke Men, Feifei Na, Wei Wang, Hanshuo Yang, Xiawei Wei

**Affiliations:** 1 Laboratory of Aging Research and Cancer Drug Target, State Key Laboratory of Biotherapy, National Clinical Research Center for Geriatrics, West China Hospital, Sichuan University, 610041 Chengdu, P.R. China; 2 Department of Oncology, Guizhou Provincial People’s Hospital, 550002 Guiyang, Guizhou P.R. China

**Keywords:** Irradiated tumor cell vaccine, Oxidized mitochondrial DNA, STING signaling, Tumour immunology, Radiotherapy

## Abstract

Exposure to ionizing radiation, a physical treatment that inactivates live tumor cells, has been extensively applied to enhance the antitumor responses induced by cancer cell vaccines in both animal research and human clinical trials. However, the mechanisms by which irradiated cells function as immunogenic tumor vaccines and induce effective antitumor responses have not been fully explored. Here, we demonstrate that oxidized mitochondrial DNA (mtDNA) and stimulator of interferon genes (STING) signaling play a key roles in the enhanced antitumor effect achieved with an irradiated tumor cell vaccine. Elevations in ROS and oxidized mtDNA 8-OHG content could be induced in irradiated tumor cells. Oxidized mtDNA derived from irradiated tumor cells gained access to the cytosol of dendritic cells (DCs). Oxidized mtDNA, as a DAMP or adjuvant, activated the STING-TBK1-IRF3-IFN-β pathway in DCs, which subsequently cross-presented irradiated tumor cell-derived antigens to CD8^+^ T cells and elicited antitumor immunity. The results of our study provide insight into the mechanism by which an irradiated cell vaccine mediates antitumor immunity, which may have implications for new strategies to improve the efficacy of irradiated vaccines.

## Introduction

As a form of immunotherapy, cancer vaccines have demonstrated objective therapeutic benefits in several randomized clinical trials.^1,2^ The activation of tumor-specific immune effects by cancer vaccines, particularly whole-tumor cell vaccines that contain all potential tumor antigens, represents a promising approach for cancer therapy.^3^ Ionizing radiation (IR), which inactivates live tumor cells to create cancer cell vaccines, has been used extensively to enhance antitumor immunity in both animal models and human clinical trials.^4–8^ In addition, local radiotherapy used alone or in combination with surgery, chemotherapy, or targeted therapy is employed to treat the primary and metastatic tumors of cancer patients.^9–11^ Furthermore, localized radiotherapy has also been shown to induce abscopal effects in several types of cancer.^12,13^ Overall, ionizing radiation can potentiate the antitumor response induced by either an irradiated tumor cell vaccine or local radiotherapy.

The potential mechanisms by which ionizing radiation enhance the antitumor response induced by either an irradiated tumor cell vaccine or local radiotherapy have been explored but remain largely unknown.^7,14–18^ It has been demonstrated that cell-surface exposure to calreticulin (CRT) is important for establishing the immunogenicity of tumor cell death elicited by irradiation, which is involved in the enhanced antitumor effect of irradiated tumor cells.^7,16^ Regarding local radiotherapy of tumors, some studies have described how local irradiation affects the tumor microenvironment. One report found that regulation of the Fas/Fas ligand pathway in tumor cells by irradiation plays an important role in their sensitization to antigen-specific CTLs (cytotoxic T lymphocytes)^17^. Another report noted that directed radiotherapy increases MHC class I expression on tumor cells, leading to increased sensitivity to antigen-specific CTLs and an immune-mediated abscopal effect.^14^ Recently, the adaptor protein STING (stimulator of interferon genes) has garnered considerable interest given its roles in sensing DNA through cGAS (cyclic GMP-AMP synthase) in DCs in response to locally irradiated tumor cells and activating the STING pathway.^15,18^ However, it remains unknown why irradiation can trigger the activation of the STING pathway. Additionally, the previous work did not address the potential role of mitochondrial DNA (mtDNA) or oxidized mtDNA in the activation of the STING pathway in response to locally irradiated tumor cells.

Recently, the concept of cell damage-associated molecular patterns (DAMPs), which are endogenous molecules released by cells undergoing abnormal cell death (e.g., during pathological insult) that are capable of activating the innate immune response,^19^ has been highlighted. Among DAMPs, mtDNA, which is similar to its bacterial ancestor, is considered an immunostimulatory agent; mtDNA consists of a circular loop and contains significant levels of unmethylated DNA in the form of CpG islands.^20^ Given that mtDNA lacks the repair proteins and protective histones found in nuclear DNA (nDNA), mtDNA is more easily attacked by reactive oxygen species (ROS),^21,22^ which are byproducts of mitochondrial respiration.^23^ The oxidized base 8-hydroxyguanosine (8-OHG), a marker of oxidative DNA damage, potentiates cytosolic immune recognition by decreasing mtDNA susceptibility to 3′ repair exonuclease 1 (TREX1)-mediated degradation.^24^ Enhanced immune sensing, which is induced by oxidized genomic DNA damaged by UV rays, is dependent on STING signaling.^24^ Another study^25^ demonstrated that herpesvirus infection induced mtDNA stress, which led to the activation of antiviral innate immune responses through the cGAS-STING pathway. In addition, oxidized mitochondrial nucleoids released by neutrophils drive type I interferon production in lupus.^26,27^ However, the role of mtDNA or oxidized mtDNA in the activation of antitumor immunity remains unexplored. Exposure of cells to ionizing radiation can cause the formation of radicals and ROS that can oxidize and damage DNA.^28^ Based on the findings mentioned above, we hypothesize that the enhanced immunogenicity of irradiated cancer cell vaccines may result from the induction of oxidized mtDNA, which in turn triggers the activation of the STING pathway.

In the present study, we demonstrate that elevations in ROS and oxidized mtDNA 8-OHG content can be induced in irradiated tumor cells, which are subsequently engulfed by DCs. We highlight the role of the oxidized mtDNA-STING pathway in the enhanced antitumor effect achieved with an irradiated cell vaccine. Our study provides insight to better understand the mechanism underlying tumor regression mediated by the irradiated cell vaccine and may have implications for new strategies to potentiate irradiated vaccine efficacy in cancer patients.

## Materials and methods

### Cell lines

A mouse colon cancer cell line (CT26), mouse Lewis lung carcinoma line (LL2) and OVA-expressing mouse thymoma cell line (EG7) were purchased from the American Type Culture Collection and maintained at 37 °C in 5% CO_2_. CT26 cells were cultured in RPMI 1640 medium (Gibco) with 10% fetal bovine serum (FBS, Invitrogen). LL2 cells were cultured in Dulbecco’s modified Eagle’s medium with 10% FBS. EG7 cells were maintained in complete RPMI 1640 medium supplemented with G418 (0.4 mg/ml; InvivoGen).

### Mice

Six-week to eight-week-old C57BL/6 or BALB/c mice purchased from Vital River (Peking, China) were maintained under specific pathogen-free conditions. *Tmem173*^*−/−*^ mice and OVA-specific T cell receptor-transgenic OT-I mice were obtained from The Jackson Laboratory. *Tlr9*^*−/−*^ mice were kindly provided by Bioindustry Division Oriental Yeast Co., Ltd. (Tokyo, Japan), and *IL-1β*^*−/−*^ mice were kindly provided by Dr. Yoichiro Iwakura of the University of Tokyo. All animal experiments were approved by the Institutional Animal Care and Use Committee of Sichuan University (Chengdu, Sichuan, China) and by the relevant animal association.

### Immunizations

To inactivate live tumor cells, cells were irradiated with X-rays (Rad Source). CT26 and LL2 cells were irradiated with 100 Gy unless otherwise indicated. EG7 cells and mtDNA derived from untreated EG7 cells were irradiated with 75 Gy unless otherwise indicated. Mice were immunized subcutaneously on the left side with 1 × 10^6^ irradiated tumor cells or irradiated tumor cells with mtDNA derived from untreated EG7 cells (5 µg per mouse) as a vaccine or PBS as a control on days 0, 14, and 21. EG7 cells were also irradiated in the presence of the ROS scavenger BHA (100 μM; Sigma)^29,30^ as a vaccine, and BHA was removed by washing after irradiation to rule out other nonspecific effects of BHA. In addition, we injected mice subcutaneously with 2000 IU of DNase I (Sigma) in 100 µl of PBS both 3 and 18 h after immunization.^31^ One week after the three immunizations, untreated live tumor cells (CT26 or LL2 cells, 1 × 10^6^; EG7 cells, 3 × 10^6^) were injected subcutaneously into the right flank of the mice, and the tumors were measured as previously described^2^ according to the following formula: tumor volume (mm^3^) = 0.52 × length × width^2^.

### Detection of cell death

To detect cell death, EG7 cells were observed under an inverted microscope (Eclipse 80i, Nikon) and were also labeled with the FITC Annexin-V Apoptosis Detection Kit with PI (#640914; BioLegend) according to the manufacturer’s instructions. EG7 cells treated with 75 Gy X-ray irradiation were cultured in vitro for 24 h. CT26 cells treated with 50 Gy X-ray irradiation were cultured in vitro for 48 h and evaluated with the FITC Annexin-V Apoptosis Detection Kit with PI by flow cytometry. For in vivo experiments, irradiated EG7 cells in the peritoneal lavage fluid were detected after 24 h. To analyze cell apoptosis, EG7 cells stained with rabbit anti-caspase-3 (1:400; #9661; Cell Signaling Technology) and FITC-goat anti-rabbit IgG (1:1000; Abcam) antibodies were evaluated on a FACSAria III flow cytometer (BD Biosciences) or visualized using a Leica TCS SP5 confocal microscope after staining with DAPI.

### Preparation of DNA and quantitative real-time PCR for mtDNA

Mice were injected intraperitoneally with 1 × 10^7^ untreated EG7 cells or irradiated EG7 cells, and PBS was used as a control. In addition, we injected mice intraperitoneally with 2000 IU of DNase I at both 3 and 18 h.^31^ Mitochondrial DNA in the peritoneal lavage fluid and cell culture supernatants collected 24 h after irradiation was concentrated and purified using the QIAamp DNA Blood Mini Kit (#51106; QIAGEN) according to the manufacturer’s instructions. The quantitation of mtDNA was performed with TaqMan probes, as previously described.^32^ Mitochondrial DNA isolation was performed using the mtDNA Isolation Kit (#ab65321; Abcam), and gDNA isolation was performed using the QIAamp DNA Blood Mini Kit (#51106; QIAGEN) according to the manufacturer’s instructions. To eliminate cellular proteins, mtDNA isolation was followed by phenol-chloroform extraction and isopropanol precipitation. The concentration of DNA was determined using a NanoDrop 2000 spectrophotometer (Thermo Scientific).

### Mitochondrial DNA depletion

Cells were treated with dideoxycytidine (ddC; 150 μM; #D5782; Sigma) for 6 days, and gDNA was extracted as described above. To measure the efficiency of mtDNA depletion, the copy number of mtDNA was normalized to that of nuclear DNA as the ratio of mtDNA encoding cytochrome c oxidase I to nuclear DNA encoding 18S ribosomal RNA by quantitative real-time PCR.^33,34^

### Generation of BMDCs in vitro

BMDCs were prepared as previously described.^15,35^ In brief, single-cell suspensions of bone marrow cells (1 × 10^6^ cells per ml) derived from C57BL/6 mice were cultured in RPMI-1640 medium containing 10% FBS, 1 mM sodium pyruvate, 20 mM HEPES, 2 mM l-glutamine, 100 U/ml penicillin G, 100 μg/ml streptomycin and 50 μM β-mercaptoethanol supplemented with mouse granulocyte-macrophage colony-stimulating factor (GM-CSF; 20 ng/ml; PeproTech). On days 3 and 5, half of the medium was replaced with fresh medium. Nonadherent BMDCs were harvested for a stimulation assay on day 7 in the presence of fresh GM-CSF. The percentage of CD11c^+^ cells in the BMDCs was evaluated by flow cytometry and found to be greater than 70%.

### Detection of cellular ROS and 8-OHG content

Intracellular ROS concentrations were determined using the ROS Assay Kit (#S0033; Beyotime). After staining, EG7 cells were immediately irradiated with X-rays, incubated for 30 min at 37 °C and analyzed by flow cytometry. To test 8-OHG oxidization induced by irradiation, EG7 cells irradiated with 75 Gy X-ray irradiation were cultured in vitro for 18 h (apoptotic EG7 cells, apoEG7 cells). The 8-OHdG levels in mtDNA and gDNA were quantified with the 8-OH-dG EIA Kit (#ab101245; Abcam) in accordance with the manufacturer’s instructions. EG7 cells were also stained with an anti-8-OHG antibody and evaluated by flow cytometry. Furthermore, to measure 8-OHG and mitochondrion content in EG7 cells, cells were stained with MitoTracker^®^ Mitochondrion-Selective according to the manufacturer’s instructions (MitoTracker^®^ Red CMXRos; #M7512; Invitrogen), a goat anti-8-OHG antibody (1:300; #ab10802; Abcam), a FITC-donkey anti-goat IgG antibody (1:1000; Thermo Fisher), and DAPI and then observed under a Leica TCS SP5 confocal microscope. Quantification of colocalization of 8-OHG and MitoTracker based on Pearson’s correlation coefficient (a perfect linear correlation is +1) was determined with ImageJ software.

### Detection of DCs in the skin

EG7 cells were injected subcutaneously. Eighteen hours later, to detect DCs in the skin, skin tissues were excised, cut into small pieces and digested with DNase I (0.1 mg/ml; Sigma) and collagenase Type IV (4 mg/ml; Gibco) for 2 h at 37 °C. The resulting cell suspensions were centrifuged, washed in PBS and passed through a 70-μm nylon mesh filter (BD Biosciences) to obtain single-cell suspensions. The single-cell suspensions were stained with PerCP-Cy^TM^5.5-conjugated anti-CD45, PE-conjugated anti-MHC-II, APC-conjugated anti-CD11c (1:100; BD Biosciences) and FITC-conjugated anti-IFN-β (1:100; PBL Assay Science) antibodies for a flow cytometry assay.

### Assays for apoptotic cell uptake

Briefly, 5-(and 6-)carboxyfluorescein diacetate succinimidyl ester (CFSE; 5 μM; #C34554; Invitrogen) cell staining was performed according to the manufacturer’s instructions. To assess uptake in vitro, irradiated CFSE-labeled apoEG7 cells were cocultured with BMDCs at a 3:1 ratio for 3 h as previously described.^36^ Then, the mixed cells were stained with antibodies against CD11c (1:100; BD Biosciences) and LIVE/DEAD^TM^ Fixable Dead Cell Stain Kits (#L34961; Thermo Fisher) according to the manufacturer’s instructions and analyzed by flow cytometry.

### Assays for oxidized mtDNA uptake

To measure 8-OHG engulfment by DCs, apoEG7 cells were cultured with BMDCs at a 1:1 ratio for 3 h in vitro.^15^ In vivo, mice were injected intraperitoneally with irradiated EG7 cells and housed for 18 h. Cells in the peritoneal lavage fluid and BMDCs stimulated with EG7 cells were stained with goat anti-8-OHG (1:300; Abcam), Alexa Fluor^®^ 647-rabbit anti-goat IgG (1:1000; Thermo Fisher), hamster anti-CD11c (1:200; #117301; BioLegend) and FITC-rabbit anti-hamster IgG (1:1000; Thermo Fisher) antibodies and observed under a Leica TCS SP5 confocal microscope after staining with DAPI. Moreover, cells in the peritoneal lavage fluid were stained with anti-CD11c, anti-CD45 and anti-8-OHG antibodies and analyzed by flow cytometry. BMDCs stimulated with EG7 cells were stained with anti-CD11c and anti-8-OHG antibodies and detected by flow cytometry.

### Immunohistochemistry

Mice were challenged with 3 × 10^6^ live EG7 cells, and EG7 tumors in mice were irradiated with 20 Gy on day 10 after tumor cell challenge.^37^ Eighteen hours after irradiation, tumor tissue was obtained, snap frozen in liquid nitrogen, embedded in ornithine carbamyl transferase medium, and sectioned. The sections were fixed in acetone, briefly air dried, and blocked with 1% BSA. The sections were then stained with goat anti-8-OHG (1:300; Abcam) and Alexa Fluor^®^ 647-rabbit anti-goat IgG (1:1000; Thermo Fisher) antibodies and observed under a Leica TCS SP5 confocal microscope.

### Western blot assay

Mitochondrial DNA isolated from EG7 cells was suspended in sterile H_2_O and treated with 75 Gy X-ray irradiation to generate oxidized mtDNA.^24^ BMDCs derived from WT mice were stimulated with 5 μg/ml mtDNA after direct irradiation or 25 μg/ml DMXAA (5,6-dimethylxanthenone-4-acetic acid; Sigma; positive control)^38^ for 3 h. Proteins were extracted with radioimmunoprecipitation assay buffer (RIPA; Beyotime) containing proteinase inhibitors (Millipore) and phosphatase inhibitors (KeyGen). Briefly, 30 μg of protein was electrophoresed in 12.5% SDS-PAGE gels and transferred to Immobilon-FL membranes (Millipore). The blots were incubated with antibodies specific for pTBK1 (Ser172), total TBK1, pIRF3 (Ser396), total IRF3 (Cell Signaling Technology) and β-actin (Zen BioScience). Quantitative analysis was performed with ImageJ software.

### Measurement of in vitro IFN-β expression

Mitochondrial DNA derived from untreated EG7 cells was irradiated with 75 Gy. BMDCs were stimulated with irradiated mtDNA or nonirradiated mtDNA for 18 h in the presence of lipofectamine (Invitrogen).^39^ Mitochondrial DNA was complexed with Lipofectamine 2000 at a ratio of 12.5 μl of lipofectamine to 2.5 μg of mtDNA. Cells in 6-well plates were stimulated with 2.5 μg of mtDNA. After an incubation, supernatants were collected, and IFN-β concentrations were measured using the VeriKine^TM^ Mouse IFN Beta ELISA Kit (#42400; PBL Assay Science) in accordance with the manufacturer’s instructions.

### OT-I T cell priming

For in vitro priming,^15,36^ lymphocytes were isolated from the spleen of OT-I mice using mouse lymphocyte separation medium according to the manufacturer’s instructions (#7211011; DAKEWE). CD8^+^ T cells were magnetically sorted using a mouse CD8^+^ T cell isolation kit (#19853; STEMCELL) in accordance with the manufacturer’s instructions. BMDCs derived from WT or STING-deficient mice were cultured with irradiated EG7 cells for 18 h (DCs:EG7 cells = 1:1), and then CD11c^+^ BMDCs were isolated using a mouse CD11c-positive selection kit (#18758; STEMCELL) according to the manufacturer’s instructions. The sorted cell purity was confirmed by flow cytometry and found to be greater than 90%. In addition to irradiation, EG7 cells were also treated with four freeze-thaw cycles using liquid nitrogen and a 37 °C water bath. OT-I CD8^+^ T cells labeled with CFSE (5 μM) were seeded in 96-well plates (10^5^ cells/well) together with CD11c^+^ BMDCs (DCs:CD8^+^ T cells = 1:5 and 1:10). Cocultures were performed for 3 days in triplicate. CFSE dilution in the CD8^+^ T cells was determined by flow cytometry.

### Flow cytometry analysis

For cell-surface staining, cells were stained with antibodies (anti-CD11c, anti-CD45, anti-MHC-II, and anti-CD8) for 30 min in PBS at 4 °C in the dark and then washed twice with PBS. For intracellular staining, cells were fixed in a 2% paraformaldehyde solution for 20 min at room temperature, permeabilized using 1% Triton X-100 for 30 min at 4 °C and washed with PBS. Then, the cells were stained with intracellular antibodies (anti-caspase-3, anti-CD8, anti-IFN-β, and anti-8-OHG) overnight at 4 °C. Data acquisition was performed on a FACSAria III flow cytometer (BD Biosciences), and the results were analyzed using FlowJo software. Further gating adjustments were made based on fluorescence minus one controls.

### Immunofluorescence assay

First, for cell-surface immunofluorescence staining, cells were stained with antibodies (hamster anti-CD11c) for 30 min in PBS at 4 °C and then washed twice with PBS. Second, for intracellular staining, cells were fixed in a 2% paraformaldehyde solution for 20 min at room temperature, blocked with 1% BSA for 30 min at 37 °C and permeabilized using 1% Triton X-100 for 30 min at 4 °C. Then, the cells were stained with intracellular antibodies (anti-caspase-3 and anti-8-OHG) overnight at 4 °C. Finally, after staining with a secondary antibody and DAPI, the cells were observed under a Leica TCS SP5 confocal microscope.

### Statistical analysis

Experiments were repeated three times. Data were analyzed using Prism 8.0 software (GraphPad Prism) and are presented as the mean ± SEM. Statistical significance was assessed using two‐tailed, unpaired Student’s *t*-tests. Survival data were analyzed using the log-rank test. *p* < 0.05 was considered significant.

## Results

### Irradiated immunogenic tumor cell vaccine mediates a protective antitumor response

**Fig. 1:**
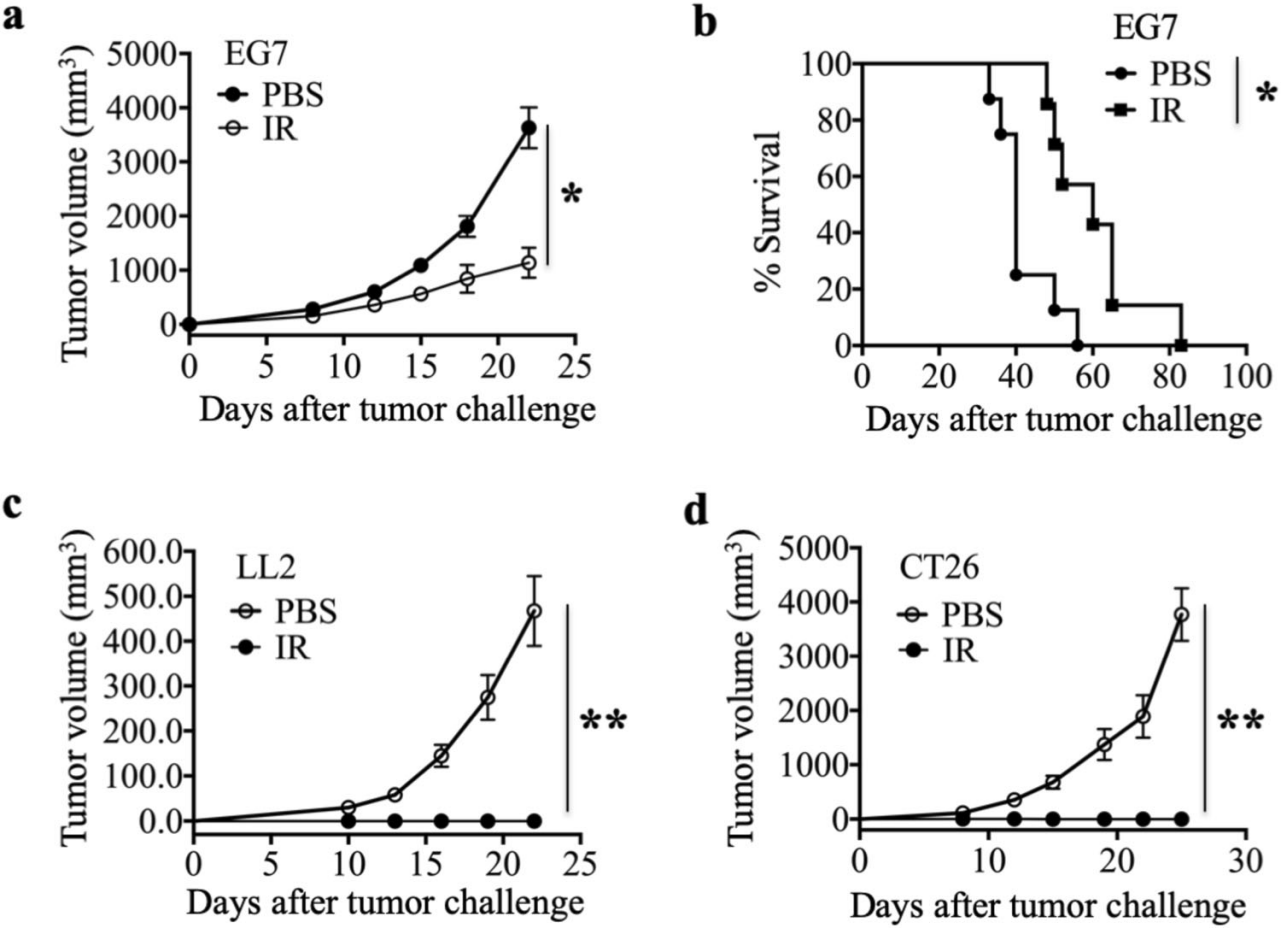
Irradiated immunogenic tumor cell vaccine mediates protective antitumor effects. **a** Growth of subcutaneous EG7 tumors in C57BL/6 mice after preventive vaccination with irradiated EG7 tumor cells (IR) or PBS. **b** Percentage survival of EG7 tumor model mice. **c**, **d** Growth of subcutaneous LL2 tumors in C57BL/6 mice (**c**) and CT26 tumor in BALB/c mice (**d**) after preventive vaccination in the LL2 and CT26 models. Representative data are shown for three (**a**–**d**) experiments conducted with 10 (**a**, **b**) or 6–8 (**c**, **d**) mice per group. Data are represented as the mean ± SEM. *p < 0.05, **p < 0.01 (Student’s *t*-test)

To investigate protective antitumor immunity, we immunized mice with an X-ray-irradiated immunogenic tumor cell vaccine or PBS on days 0, 14, and 21 and challenged them with tumor cells on day 7 after the third immunization. Tumor growth inhibition was determined by measuring tumor size. The irradiated immunogenic tumor cell vaccine demonstrated apparent and persistent protection against tumor growth in different tumor models established with three different mouse strains (Fig. 1a–d), including OVA-expressing mouse thymoma cells (EG7), mouse Lewis lung carcinoma cells (LL2) and mouse colon cancer cells (CT26). In the EG7 T cell lymphoma model, the survival time of the vaccine group was significantly longer than that of the PBS group (Fig. 1b). In addition, in the CT26 and LL2 models, the percentages of tumor-free mice in the irradiated tumor cell vaccine group was 100%, but the group immunized with PBS had 100% tumor-bearing mice (Fig. 1c, d). However, the potential mechanisms by which ionizing radiation enhances the antitumor response of the irradiated tumor cell vaccine remain largely unknown. We hypothesize that the oxidized mtDNA-STING pathway plays an important role in the enhanced immunogenicity of irradiated cancer cell vaccines.

### Irradiation induces cell apoptosis, and the antitumor effect does not correlate with mtDNA leakage from irradiated cells

**Fig. 2:**
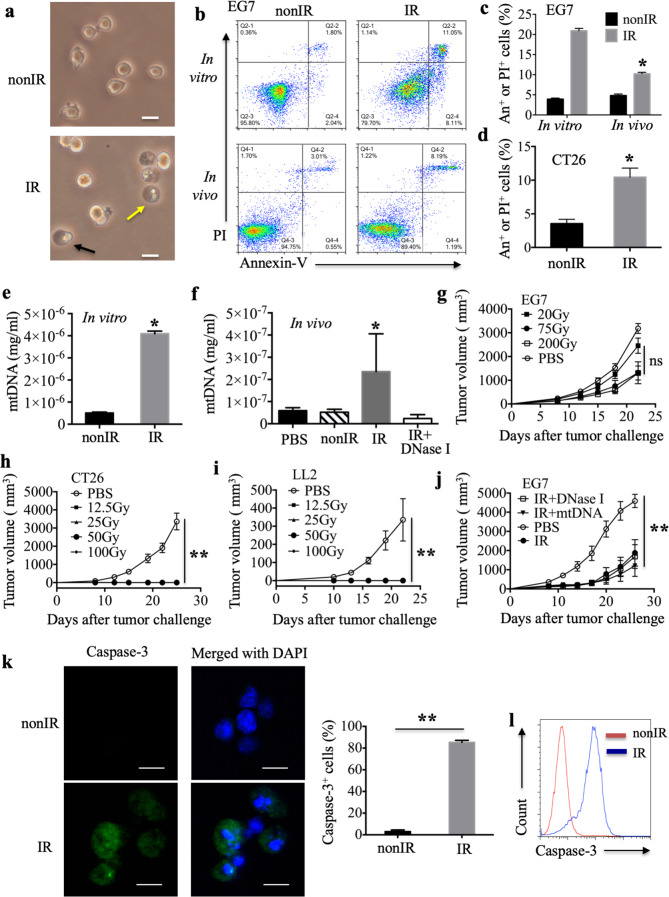
Irradiation induces cell apoptosis, and the antitumor response induced by the irradiated tumor cell vaccine does not correlate with mtDNA leakage from irradiated cells. **a** The morphological changes in untreated EG7 cells (nonIR) and irradiated EG7 cells (IR); scale bar, 20 μm. **b** A representative experiment for the detection of untreated EG7 cells and dead EG7 cells induced by irradiation after 24 h in vitro or in vivo, as determined by flow cytometry analysis of Annexin-V and PI staining. **c**, **d** Percentages of Annexin-V^+^ or PI^+^ cells in all cells in the EG7 model (**c**) and CT26 model (**d**). **e**, **f** Quantity of free mtDNA in supernatants derived from untreated EG7 cells or irradiated EG7 cells in vitro (**e**) and in the peritoneal lavage fluid of mice treated intraperitoneally with PBS, untreated EG7 cells, irradiated EG7 cells or irradiated EG7 cells with DNase I (**f**). **g** Growth of subcutaneous EG7 tumors in C57BL/6 mice after preventive vaccination with EG7 cells irradiated with different X-ray doses or PBS. **h**, **i** Growth of subcutaneous CT26 tumors in BALB/c mice (**h**) and LL2 tumor in C57BL/6 mice (**i**) after preventive vaccination with tumor cells irradiated with different X-ray doses or PBS. **j** Preventive vaccination with irradiated EG7 cells combined with mtDNA derived from untreated EG7 cells or treated with DNase I. **k** A representative immunofluorescence experiment with staining for Caspase-3 (green) and cell nuclei (blue) in untreated EG7 cells and irradiated EG7 cells; scale bar, 10 μm. The percentage of Caspase-3^+^ EG7 cells in total DAPI-positive nuclei was calculated over ten 600× fields. **l** Caspase-3 detected by flow cytometry. Representative data are shown for three (**a**–**l**) experiments conducted with 6–9 (**g**–**j**) mice per group. Data are represented as the mean ± SEM. *p < 0.05, **p < 0.01, ns not significant (Student’s *t*-test)

Intracellular molecules secreted, released and/or exposed by dying, injured or stressed cells, which are referred to as DAMPs, act as a danger signals to alert the immune system to abnormal cell injury or death.^19^ Our laboratory^32^ demonstrated that cell necrosis could be induced by cationic nanocarriers and the resulting leakage of mtDNA activated an innate immune response in vivo. It is conceivable that the free mtDNA released from irradiated dead cells as a DAMP is associated with the antitumor response induced by an irradiated cell vaccine. To test this hypothesis, we first examined tumor cell death induced by irradiation. EG7 cells treated with 75 Gy X-ray irradiation exhibited morphological changes characteristic of necrotic cells (cytoplasmic swelling, indicated by black arrow) and apoptotic cells (cell fragments, indicated by yellow arrow) (Fig. 2a). The treatment also led to an apparent increase in the number of necrotic cells and apoptotic cells, as detected by flow cytometry with Annexin-V and PI staining (Fig. 2b). PI-positive or Annexin-V-positive regions, which are recognized as dead cells, markedly increased from 4.2–20.3% in vitro and from 5.2 to 10.6% in the peritoneal lavage fluid, as detected 24 h after X-ray treatment in the EG7 model (Fig. 2c). Irradiation could also mediate cell death in an in vitro CT26 model, as determined by flow cytometry (Fig. 2d).

Next, we detected mtDNA leakage into the culture medium supernatant of irradiated EG7 cells by real-time quantitative PCR. Irradiation increased the concentration of free mtDNA (Fig. 2e). Release of mtDNA into the peritoneal lavage fluid was also confirmed 24 h after the injection of irradiated EG7 cells, and DNase I could degrade free mtDNA (Fig. 2f). Furthermore, we hypothesized that the X-ray dose affects the amount of mtDNA released from dead cells, subsequently modulating the antitumor effects. Thus, an X-ray dose-escalation study to investigate the effect of the irradiated tumor cell vaccine was performed. However, no differences in tumor volume after immunization with irradiated tumor cell vaccines created with different X-ray doses were noted in the three tumor models (Fig. 2g–i). All the tumor cell vaccines irradiated with a dose between 12.5 Gy and 100 Gy had 100% tumor-free mice in the CT26 and LL2 models (Fig. 2h, i). In the EG7 model, although it appeared that 20 Gy was not different from the other doses, the difference was not statistically significant (*p* > 0.05) (Fig. 2g). Mice were immunized subcutaneously in the left side with the irradiated tumor cell vaccine, and we measured the tumors in the right flank of the immunized mice after tumor challenge. However, tumors were noted in the left side from day 12 to day 22 in 3/9 mice after immunization with the irradiated EG7 cell vaccine created with 20 Gy, which did not occur with the vaccines created with 75 or 200 Gy (data not shown). This result demonstrates that irradiation with 20 Gy is insufficient to inactivate EG7 tumor cells, which increases the tumor burden and may affect the antitumor response induced by the irradiated EG7 vaccine (Fig. 2g). Moreover, treatment of mice with DNase I after immunization failed to prevent the antitumor activity induced by the irradiated vaccine (Fig. 2j). The addition of purified mtDNA derived from untreated EG7 cells did not potentiate the antitumor activity induced by the irradiated tumor cell vaccine (Fig. 2j). We deduced that free mtDNA was minimally released from cells undergoing apoptosis, which is the major type of cell death occurring after irradiation because preservation of the cell membrane during apoptosis results in limited stimulation of host immune cells. The induction of cell apoptosis by radiotherapy was further confirmed in the present study. Apoptotic markers of caspase activation (caspase-3-p20 immunoreactivity) and apoptotic fragmented nuclei appeared in the cytoplasm of irradiated cells after X-ray irradiation (Fig. 2k). The percentage of caspase-3-positive EG7 cells among the total population of cells with a DAPI-positive nucleus induced 24 h after irradiation was increased in the irradiated group compared with the nonirradiated group (Fig. 2k), and this finding was also evident by flow cytometry (Fig. 2l). One previous study^15^ demonstrated that DNA from irradiated tumor cells is sensed by host cGAS during a cell–cell contact-mediated process and DCs do not engulf free DNA fragments, which was confirmed by the application of a physical barrier, an actin polymerization inhibitor and DNase I. Therefore, it is possible that mtDNA leakage from irradiated cells does not correlate with the antitumor response elicited by the irradiated cell vaccine.

### Oxidative damage of mtDNA after irradiation

**Fig. 3:**
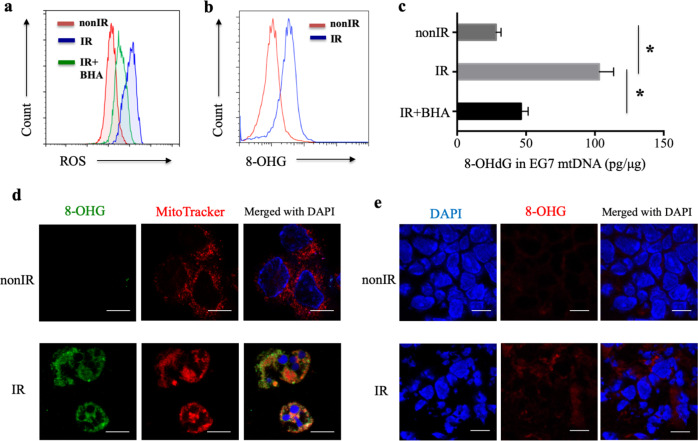
Oxidative damage to mtDNA after irradiation. **a** EG7 cells were treated with X-ray irradiation in the presence of the ROS scavenger BHA (BHA-plus EG7). ROS production in EG7 cells was measured using the dye DCF. **b** 8-OHG in EG7 cells was detected by flow cytometry. **c** The concentration of 8-OHdG (pg) in mtDNA (μg) isolated from EG7 cells was measured with a competitive 8-OHdG EIA kit. **d** Immunofluorescence staining with an anti-8-OHG antibody (green), MitoTracker (red), and DAPI (cell nuclei, blue) was performed with untreated EG7 cells and apoEG7 cells; scale bar, 10 μm. **e** Immunofluorescence staining for 8-OHG (red) and cell nuclei (DAPI, blue) in untreated EG7 tumor sections and irradiated EG7 tumor sections was performed; scale bar, 10 µm. Data from three independent experiments were pooled and are shown as the means ± SEM. *p < 0.05 (Student’s *t*-test)

To explore the role of oxidized mtDNA in the activation of the antitumor effect by the irradiated tumor cell vaccine, oxidative damage experiments were performed. ROS production was markedly increased after X-ray irradiation (Fig. 3a). Butylated hydroxyanisole (BHA), a ROS scavenger,^29,40^ demonstrated the ability to decrease the ROS levels induced by irradiation in our study (Fig. 3a). ROS are highly reactive with DNA and induce distinct DNA modifications. The oxidation of guanine to 8-OHG (8-hydroxyguanosine), a hallmark of oxidative DNA damage,^41^ was increased in EG7 cells treated with 75 Gy X-ray irradiation after 18 h (apoptotic EG7, apoEG7 cells) compared with untreated EG7 cells, as detected by flow cytometry (Fig. 3b). Moreover, the 8-hydroxy-2-deoxyguanosine (8-OHdG) content in mtDNA was enhanced in irradiated apoEG7 cells but was decreased by BHA, as measured using an 8-OHdG EIA kit (Fig. 3c). Unlike nuclear DNA (nDNA), mtDNA is not protected by histones. Therefore, the proofreading capacity is limited, and mtDNA is more easily attacked by ROS.^22^ Indeed, apoEG7 cells exhibited intense labeling in the peripheral nuclear region with minimal 8-OHG in the central area in our study (Fig. 3d), which was also demonstrated in irradiated tumor sections from an in vivo experiment (Fig. 3e). Moreover, to label mitochondria, cells were incubated with MitoTracker^®^ probes, the signal for oxidized 8-OHG colocalized with MitoTracker fluorescence, and the Pearson’s correlation coefficient was +0.60 (a perfect linear correlation is +1) (Fig. 3d), confirming that most 8-OHG oxidation induced by X-ray irradiation occurred in mitochondrial DNA rather than in chromosomal DNA.

### Oxidized mtDNA from irradiated cells gains access to the cytoplasm of DCs

**Fig. 4:**
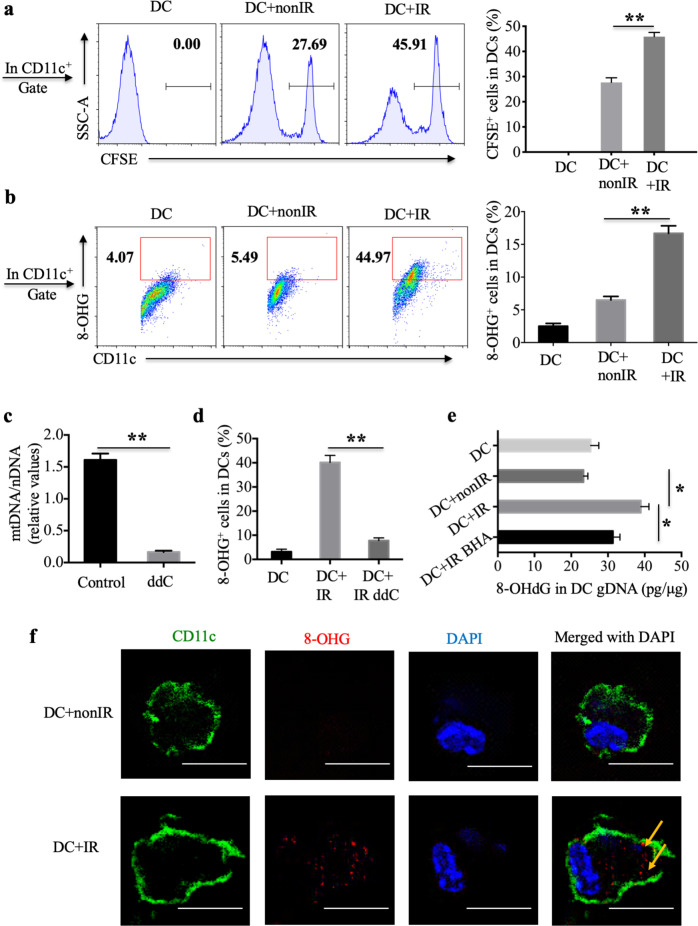
Oxidized mtDNA of irradiated cells gains access to the cytoplasm of DCs in vitro. **a** BMDCs were cultured with nonirradiated CFSE-stained EG7 cells or irradiated CFSE-stained apoEG7 cells for 3 h in vitro, subsequently labeled with an anti-CD11c antibody, and detected by flow cytometry. The percentages of CD11c^+^CFSE^+^ cells in CD11c^+^ DCs are shown. **b** BMDCs were cultured with untreated EG7 cells or apoEG7 cells for 3 h (stimulated DCs). The percentages of CD11c^+^8-OHG^+^ cells in CD11c^+^ DCs were determined by flow cytometry. **c** EG7 cells were treated with dideoxycytidine (ddC, 150 μM) for 6 days (ddC-plus EG7). The ratio of mitochondrial DNA to nuclear DNA was measured by real-time PCR, and untreated EG7 cells were used as a control. **d** BMDCs were cultured with ddC-plus apoEG7 cells for 3 h, subsequently labeled with anti-CD11c and anti-8-OHG antibodies and detected by flow cytometry. **e** Genomic DNA (μg) isolated from CD11c^+^ cells purified from stimulated DCs was evaluated with the 8-OHdG (pg) EIA Kit. BMDCs were also cultured with BHA-plus apoEG7 cells for 3 h. **f** Stimulated DCs were labeled to visualize CD11c (green), 8-OHG (red), and cell nuclei (DAPI, blue); scale bar, 10 μm. Data are representative of three experiments and presented as the mean ± SEM. *p < 0.05, **p < 0.01 (Student’s *t*-test)

To investigate whether oxidative mtDNA from irradiated tumor cells is delivered to the cytosol of DCs, irradiated CFSE-labeled apoEG7 cells were cocultured with BMDCs (bone marrow-derived DCs) for 3 h. We found that BMDCs engulfed more irradiated CFSE-labeled apoEG7 cells than nonirradiated EG7 cells in vitro (Fig. 4a and supplementary Fig. S1a). We also observed that approximately 45% of CD11c^+^ DCs showed positive staining for oxidized 8-OHG derived from irradiated EG7 tumor cells, whereas controls showed only 4–5% positive staining, as assessed by flow cytometry (Fig. 4b). Furthermore, we confirmed the result with an independent protocol for mtDNA depletion with dideoxycytidine (ddC), an inhibitor of mtDNA polymerase γ that does not affect the functions of nuclear DNA polymerases.^42,43^ We cultured EG7 tumor cells with dideoxycytidine (ddC), which efficiently depleted mtDNA from EG7 cells (Fig. 4c) and decreased the amounts of oxidized mtDNA ingested by DCs, as detected by flow cytometry (Fig. 4d). Moreover, BMDCs were cultured with apoEG7 cells for 3 h, and genomic DNA (gDNA) isolated from purified CD11c^+^ BMDCs was evaluated using an 8-OHdG EIA kit. Similarly, higher levels of 8-OHdG in gDNA were noted in BMDCs cocultured with apoEG7 cells than in those cocultured with nonirradiated EG7 cells or BHA-plus apoEG7 cells (Fig. 4e). To further confirm whether 8-OHG derived from apoEG7 cell mtDNA gains access to the cytoplasm of DCs, BMDCs were cultured with apoEG7 cells for 3 h and evaluated by immunocytochemistry. As noted for the irradiation group, oxidized mtDNA derived from irradiated EG7 cells was detected in the cytoplasm of DCs (Fig. 4f).

**Fig. 5:**
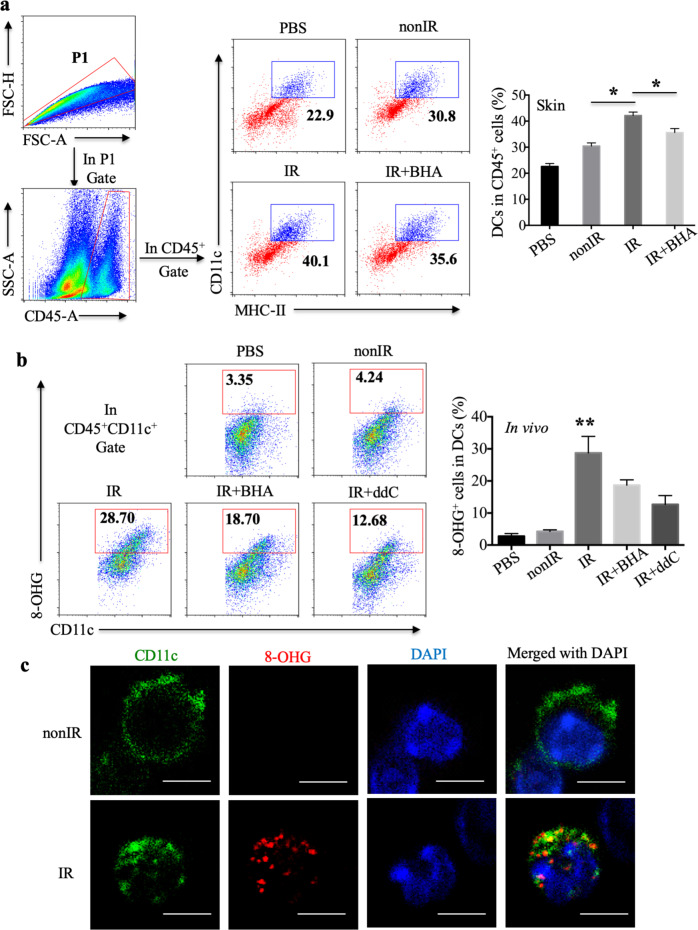
Oxidized mtDNA derived from irradiated tumor cells is transferred to host DCs in vivo. **a** Skin cells isolated from mice 18 h after subcutaneous injection of PBS, untreated EG7 cells, irradiated EG7 cells or BHA-plus irradiated EG7 cells were analyzed by flow cytometry. The percentages of CD45^+^CD11c^+^MHCII^+^ DCs among CD45^+^ cells in the skin are shown. **b** Cells in the peritoneal lavage fluid of mice treated intraperitoneally with PBS, untreated EG7 cells, irradiated EG7 cells, BHA-plus EG7 or ddC-plus EG7 (stimulated DCs in vivo) were labeled with anti-CD45, anti-CD11c and anti-8-OHG antibodies and assessed by flow cytometry (*n* = 3). **c** Stimulated DCs in vivo were labeled to visualize CD11c (green), 8-OHG (red) and cell nuclei (DAPI, blue); scale bar, 5 μm. Representative data are shown for three experiments conducted with 3–5 mice per group. Data are represented as the mean ± SEM. *p < 0.05, **p < 0.01 (Student’s *t*-test)

Moreover, 18 h after subcutaneous immunization with irradiated EG7 cells, elevated amounts of CD45^+^CD11c^+^MHC-II^+^ DCs among CD45^+^ leukocytes were found in the mouse skin (Fig. 5a). The ROS scavenger BHA reduced the percentage of DCs recruited to the skin (Fig. 5a). To further assess whether oxidized mtDNA was transferred to DCs in vivo, mice were treated intraperitoneally with irradiated EG7 cells and housed for 18 h. The percentage of CD45^+^CD11c^+^8-OHG^+^ cells in CD45^+^CD11c^+^ DCs in the peritoneal lavage fluid was markedly increased after treatment with irradiated EG7 cells compared with treatment with nonirradiated EG7 cells or PBS (Fig. 5b and Supplementary Fig. S1b). Moreover, both BHA and ddC decreased the amount of oxidized mtDNA ingested by DCs in vivo (Fig. 5b). Furthermore, as observed for the irradiation group, oxidized mtDNA derived from irradiated EG7 cells could be detected in the cytoplasm of DCs in vivo by immunocytochemistry (Fig. 5c). Collectively, these in vitro and in vivo data suggest that oxidized mtDNA gains access to the cytosol of DCs.

### Oxidized mtDNA activates the STING pathway in DCs, leading to CD8^+^ T cell proliferation

**Fig. 6:**
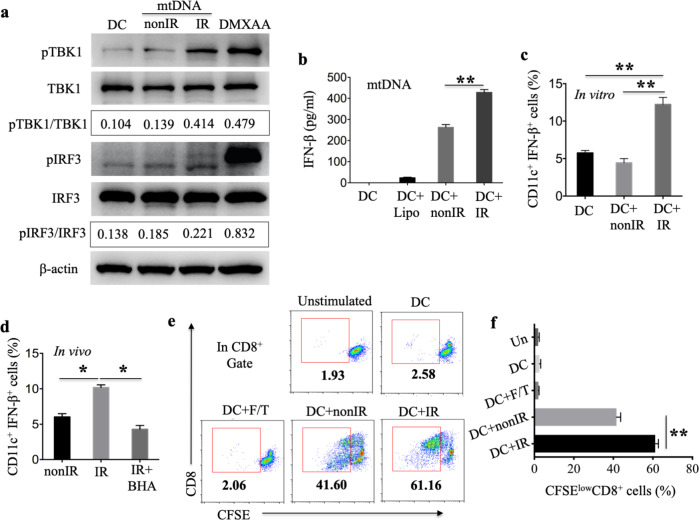
Oxidized mtDNA ingested by DCs activates the TBK1-IRF3-IFN-β pathway in DCs, which leads to CD8+ T cell proliferation. **a** Mitochondrial DNA isolated from EG7 cells was directly irradiated with X-rays. BMDCs were subsequently stimulated with mtDNA for 3 h, and the amounts of pTBK1, total TBK1, pIRF3, total IRF3, and β-actin were measured by immunoblotting. DMXAA was used as a positive control. **b** BMDCs were stimulated with directly irradiated mtDNA for 18 h in the presence of lipofectamine, and the supernatants were collected to measure IFN-β levels by ELISA. **c** BMDCs, which were cultured with untreated EG7 cells or apoEG7 cells for 3 h, were labeled with anti-CD11c and anti-IFN-β antibodies and evaluated by flow cytometry. The percentages of CD11c^+^IFN-β^+^ cells in CD11c^+^ BMDCs are shown. **d** Skin cells collected as described in Fig. 5a were labeled with anti*-*CD11c, anti-CD45 and anti-IFN-β antibodies and assessed by flow cytometry. The percentages of CD45^+^CD11c^**+**^IFN-β^+^ DCs among CD45^+^ cells are shown (*n* = 3). **e**, **f** EG7 cells treated with irradiation or freeze-thaw cycles (F/T) were cultured with BMDCs from WT mice for 18 h (loaded DCs). Subsequently, CD11c^+^ cells purified from the loaded DCs were incubated with sorted CFSE-stained CD8^+^ T cells from OT-I mice (DCs:CD8^+^ T cells = 1:5). We estimated the proliferation of the CD8^+^ T cells by measuring the percentages of CFSE^low^CD8^+^ T cells in CD8^+^ T cells by flow cytometry. Data are representative of three experiments and presented as the mean ± SEM. *p < 0.05, **p < 0.01 (Student’s *t*-test)

Oxidized DNA modified with 8-OHG after UV irradiation is resistant to cytosolic exonuclease TREX-1-mediated degradation, and STING-dependent signaling but not TLR9 signaling mediates the type I IFN response triggered by UV irradiation-damaged DNA.^24^ To validate the role of oxidized mtDNA in STING signaling activation, mtDNA isolated from EG7 cells and directly exposed to X-ray irradiation was used to stimulate BMDCs. TANK-binding kinase 1 (TBK1) phosphorylation and IRF3 (IFN regulatory factor 3) phosphorylation in BMDCs were detected by immunoblot analysis. We observed increased phosphorylation of TBK1 and IRF3 in BMDCs stimulated with irradiated oxidized mtDNA compared with those stimulated with nonirradiated mtDNA. The amount of each protein was normalized to that of the β-actin loading control, and the ratio of phosphorylated protein to total protein was quantified (pTBK1/TBK1: IR [0.414] versus nonIR [0.139], versus unstimulated DCs [0.104], *p* < 0.01; pIRF3/IRF3: IR [0.221] versus nonIR [0.185], versus unstimulated DCs [0.138], *p* < 0.05) (Fig. 6a). STING is a major regulator of type I IFN induction by intracellular exogenous pathogen-derived DNA in a TLR-independent manner.^44,45^ To test whether the oxidized mtDNA-IRF3-STING pathway is responsible for type I IFN induction following irradiation, an in vitro experiment utilizing mtDNA after exposure to direct irradiation or nonirradiated mtDNA combined with lipofectamine showed that the irradiated mtDNA provoked greater IFN-β production by BMDCs (Fig. 6b). Moreover, we measured the percentage of BMDCs that demonstrated IFN-β secretion after coculturing BMDCs with apoEG7 cells for 3 h. There were more CD11c^+^IFN-β^+^ cells among CD11c^+^ BMDCs in the irradiation groups than the nonirradiated EG7 cell groups, as assessed by flow cytometry (Fig. 6c). We also measured the percentage of CD45^+^CD11c^+^IFN-β^+^ DCs in the skin 18 h after subcutaneous immunization with irradiated EG7 cells. As shown in Fig. 5a, relatively high numbers of CD45^+^CD11c^+^MHC-II^+^ DCs and CD45^+^CD11c^+^IFN-β^+^ DCs among all CD45^+^ cells were recruited into the mouse skin (Fig. 6d).

To map whether the functional capability of DCs to cross-present antigen is potentiated by stimulation with irradiated tumor cells compared with stimulation with nonirradiated tumor cells, we treated EG7 cells with irradiation, freeze-thaw cycles or apoEG7 cells with the addition of BHA. EG7 cells were cultured with BMDCs derived from WT mice for 18 h (loaded BMDCs). Subsequently, sorted CD11c^+^ BMDCs isolated from the loaded BMDCs were incubated with CFSE-stained purified CD8^+^ T cells derived from OT-I mice for 3 days. We estimated the proliferation of the CD8^+^ T cells by measuring the percentage of CFSE^low^CD8^+^ T cells among all CD8^+^ T cells by flow cytometry. BMDCs alone or BMDCs stimulated with a freeze-thaw lysate failed to induce CD8^+^ T cell proliferation, but BMDCs treated with EG7 cells could induce the proliferation of OVA-specific CD8^+^ T cells (Fig. 6e, f and supplementary Fig. S2a). Furthermore, the proliferation induced by loaded BMDCs stimulated with irradiated EG7 (apoEG7) cells was increased compared with that induced by BMDCs treated with nonirradiated EG7 cells (Fig. 6e, f). In conclusion, oxidized mtDNA ingested by DCs activates the TBK1-IRF3-IFN-β pathway in DCs, leading to CD8^+^ T cell proliferation.

### The oxidized mtDNA-STING pathway plays key roles in the enhanced antitumor response achieved with an irradiated cell vaccine

**Fig. 7:**
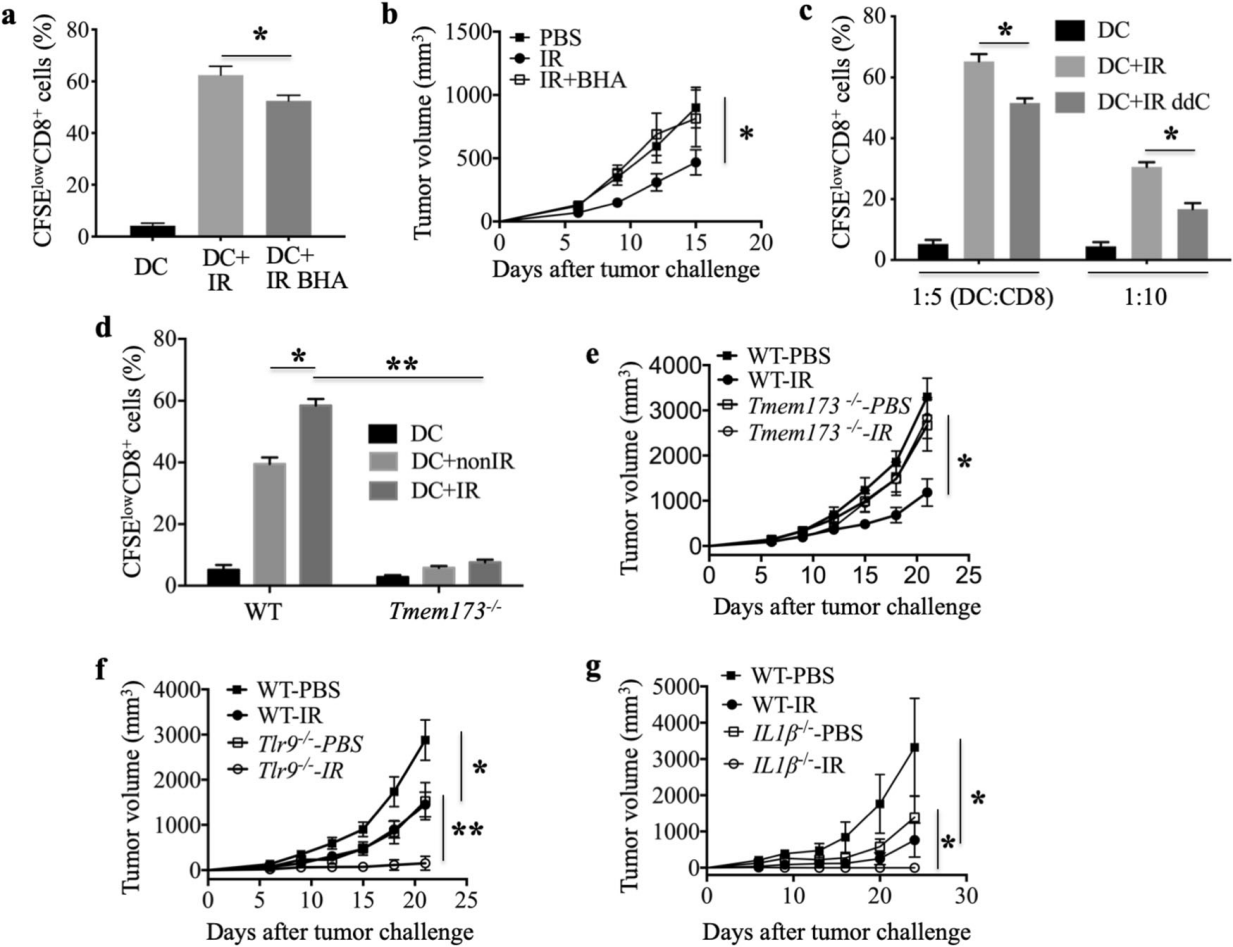
The oxidized mtDNA-STING pathway is required for the antitumor effect of the irradiated tumor cell vaccine. **a**, **c** As described in Fig. 6e, EG7 cells treated with BHA-plus irradiation (**a**) or ddC-plus irradiation (**c**) were cultured with BMDCs from WT mice (WTDCs) for 18 h. In **c** the ratio of DCs to CD8^+^ T cells is 1:5 or 1:10. The ratio of DCs to CD8^+^ T cells was 1:5 in **a**, **d**, Fig. 6e. We estimated the proliferation of CD8^+^ T cells by measuring the percentages of CFSE^low^CD8^+^ cells in CD8^+^ T cells by flow cytometry. **b** The growth of subcutaneous EG7 cells after preventive vaccination with PBS, irradiated EG7 cells or BHA-plus irradiated EG7 cells was monitored. **d** The proliferation of CD8^+^ T cells cultured with WTDCs or *Tmem173*^*−/−*^ DCs is shown. *Tmem173*^*−/−*^ represents STING-deficient mice. **e** Tumor growth was monitored in WT and *Tmem173*^*−/−*^ mice after preventive vaccination with PBS or irradiated EG7 cells. **f** Tumor growth was monitored in WT and *Tlr9*^*−/−*^ mice after vaccination. **g** Tumor growth was monitored in WT and *IL-1β*^*−/−*^ mice after vaccination. Representative data are shown for three (**a**–**g**) experiments conducted with 6–8 (**b**, **e**–**g**) mice per group. Data are represented as the mean ± SEM. *p < 0.05, **p < 0.01 (Student’s *t*-test)

To further explore the role of oxidized mtDNA-STING signaling in the enhanced antitumor effect triggered by the irradiated cell vaccine, in an OT-I T cell priming experiment, we observed that the proliferation of CD8^+^ T cells induced by loaded BMDCs treated with apoEG7 cells with the addition of BHA was decreased compared with that induced by BMDCs stimulated with apoEG7 cells (Fig. 7a and Supplementary Fig. S2b). We also found that treatment of irradiated EG7 cells with the ROS scavenger BHA significantly impaired the irradiated EG7 cell vaccine-mediated antitumor effect (Fig. 7b), demonstrating that oxidative damage is important for the antitumor effect induced by the irradiated tumor cell vaccine. Furthermore, ddC, an inhibitor of mtDNA polymerase, γ, also had the ability to impair the proliferation of CD8^+^ T cells activated by irradiated EG7 cells (Fig. 7c and Supplementary Fig. S2c). In addition, the absence of host STING prevented the induction of CD8^+^ T cell proliferation activated by irradiated EG7 cells in the OT-I T cell priming experiment (STING is encoded by *Tmem173*) (Fig. 7d). Moreover, EG7 cells were implanted into the flanks of WT (wild-type) and STING-deficient mice after three protective immunizations with an X-ray-irradiated EG7 cell vaccine, and tumor growth was monitored. We again found that the absence of host STING significantly impaired the antitumor effect of the cell vaccine (Fig. 7e), demonstrating that STING is required for the CD8^+^ T cell proliferation activated by irradiated EG7 cells and is important in the irradiated tumor cell vaccine eliciting effective antitumor immunity.

However, TLR9 (Toll-like receptor 9) and NLRP3 (Nod-like receptor-family pyrin domain containing 3) may also play roles in innate immune signaling by recognizing intracellular DNA, such as mitochondrial DNA.^46^ To investigate whether TLR9 is required to mediate the antitumor response induced by the irradiated cell vaccine, EG7 tumor cells were implanted in the flanks of WT and *Tlr9*^*−/−*^ mice after three immunizations with the irradiated EG7 cell vaccine. Tumor growth inhibition was comparable between the WT and *Tlr9*^*−/−*^ mice (Fig. 7f), demonstrating that host TLR9 may not be required for the antitumor effect induced by the irradiated vaccine. In addition, IL-1β expression induced by AIM2 (absent in melanoma 2), TLR9 and NLRP3 plays an important role in the immune response to DNA.^46,47^ To examine whether IL-1β is involved in the antitumor effect of the vaccine, EG7 cells were implanted in WT and *IL-1β*^−/−^ mice after immunizations. The absence of host IL-1β also did not affect the antitumor activity induced by the irradiated vaccine (Fig. 7g), suggesting that IL-1β secretion may not be required. Taken together, these results demonstrate that oxidized mtDNA-STING signaling but not TLR9 signaling or IL-1β secretion is involved in the protective antitumor immunity induced by the irradiated cancer cell vaccine.

## Discussion

**Fig. 8:**
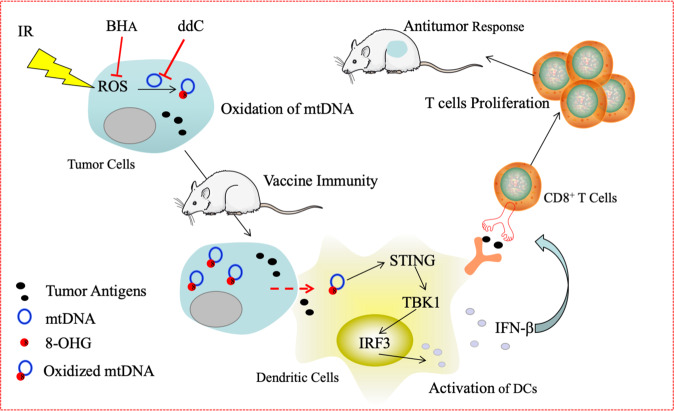
Oxidized mitochondrial DNA from irradiated tumor cells gains access to the cytoplasm of dendritic cells, subsequently activating the STING-TBK1-IRF3-type I interferon pathway and eliciting antitumor immunity. Irradiation induces tumor cell apoptosis, ROS release, and upregulation of the 8-OHG content, which results in oxidative mtDNA damage in tumor cells. After immunization with an irradiated tumor cell vaccine, oxidized mtDNA derived from tumor cells is transferred to the cytoplasm of DCs, which in turn activates STING-TBK1-IRF3 signaling in the DC cytoplasm and induces type I interferon production. Type I interferons enhance the cross-presentation of apoptotic tumor cell-derived antigens after irradiation and potentiate the proliferation of CD8^+^ T cells, leading to protective antitumor immunity. Moreover, a ROS scavenger (BHA) and an inhibitor of mtDNA polymerase γ (ddC) have the ability to impair CD8^+^ T cell proliferation by inhibiting oxidative mtDNA damage

Ionizing radiation, a physical treatment to inactivate live tumor cells, has been extensively applied to enhance the antitumor effects of cancer cell vaccines in both animal research and human clinical trials.^4–8^ Nevertheless, the mechanisms by which irradiated cells function as an immunogenic tumor vaccine and induce an effective antitumor response have not been fully explored. In the present study, we found that the mtDNA-STING pathway was important for the protective antitumor effect of an irradiated cancer cell vaccine, while TLR9 and IL-1β signaling was not. X-ray irradiation induced tumor cell apoptosis and functioned as an effective vaccination strategy via lethal stimulation. In addition, ROS induced by X-ray irradiation produced 8-OHG, resulting in oxidative mtDNA damage in EG7 cells. The oxidation of 8-OHG protects oxidized DNA strands from TREX1 degradation.^24^ Moreover, DCs engulfed greater numbers of irradiated EG7 cells than untreated EG7 cells. Oxidized mtDNA derived from irradiated tumor cells gained access to the cytosol of DCs, which activated STING-TBK1-IRF3 signaling in the DC cytoplasm. STING is a critical mediator of IFN-β induction mediated by intracellular exogenous DNA in DCs, a type of antigen-presenting cell (APC), thus enhancing the cross-presentation of apoptotic EG7 cell-derived antigens after irradiation. Moreover, loaded DCs cocultured with apoEG7 cells potentiated the proliferation of CD8^+^ T cells after stimulation in a STING pathway-dependent manner. We also observed that the ROS scavenger BHA inhibited the antitumor effect of the irradiated tumor cell vaccine by impairing oxidative mtDNA damage and CD8^+^ T cell proliferation. Based on our findings, we demonstrate that the elevations in ROS and mtDNA 8-OHG content can be induced within irradiated tumor cells and oxidized mtDNA is transferred to the cytoplasm of DCs. Furthermore, the STING pathway in DCs, which is activated by oxidized mtDNA, is critical for eliciting the antitumor immunity mediated by the irradiated tumor cell vaccine (Fig. 8).

In the present study, we observed that mtDNA, i.e., a DAMP, played an important role in the protective antitumor response induced by the irradiated immunogenic tumor cell vaccine. However, the application of different X-ray doses, which may potentially result in different amounts of mtDNA leakage from the irradiated cells, did not correlate with the antitumor response induced by the irradiated cell vaccine. It is possible that the intact cell membrane retained during apoptosis, which is the major type of cell death after irradiation, results in minimal mtDNA leakage and limited stimulation of host immune cells. In addition, X-rays induce tumor cells to generate elevated amounts of ROS, which results in subsequent oxidative DNA damage (i.e., 8-OHG), and these data are consistent with previous data obtained with UV irradiation.^24^ Given that mtDNA lacks the repair proteins and protective histones found in nDNA, mtDNA is more easily attacked by ROS,^22^ which was confirmed after exposure of cells to 5 Gy gamma-ray irradiation.^21^ In our study, we found that X-ray-irradiated EG7 tumor cells exhibited intense labeling in the peripheral nuclear region with minimal 8-OHG content in the central area in vitro and in vivo and that oxidized 8-OHG colocalized with MitoTracker fluorescence, which labeled mitochondria. In addition, the 8-OHdG content in mtDNA derived from apoEG7 cells was markedly increased. Cell-surface exposure of CRT is critical for establishing the immunogenicity of tumor cell death elicited by irradiation, which is involved in the enhanced phagocytosis of irradiated cells by DCs.^7,16^ Our results revealed that DCs could engulf more irradiated EG7 cells than untreated EG7 cells and that oxidized mtDNA was delivered to the cytoplasm of DCs after irradiated tumor cell vaccination and subsequently elicited antitumor immunity. Our study does not exclude the possibility that other DAMPs, such as other mitochondrial components (proteins, peptides, cytochrome c, ATP, etc.), or nDNA can efficiently activate the STING pathway, but it demonstrates that mtDNA is a major initiator of the maximal antitumor effect induced by the irradiated cell vaccine. Moreover, DNA vaccines have emerged as an attractive immunotherapeutic strategy against cancer due to their simplicity, stability, and safety.^48^ It is possible to design oxidized mtDNA for use as an adjuvant to enhance the antitumor efficacy of cancer vaccines, including DNA vaccines, cell vaccines, and DC vaccines.

Our study found that the mtDNA-STING pathway is critical for potentiating the effective antitumor immunity triggered by an irradiated cell vaccine. The mechanisms by which ionizing radiation can potentiate the antitumor response induced by either an irradiated tumor cell vaccine or local radiotherapy have been explored but remain largely unknown.^12,14–17^ It has been reported that the cytosolic DNA-STING pathway and canonical NF-κB pathway play important roles in the response to local irradiation.^15^^[,18^ However, these studies did not address the potential role of mitochondrial DNA or oxidized mitochondrial DNA in the activation of the STING pathway in response to locally irradiated tumor cells. Our research is focused on an irradiated cell vaccine, not local irradiation. However, the mechanism underlying the response to local irradiation mentioned above may be partially involved in the response elicited by our irradiated cell vaccine. The host protein STING has been identified as a central signaling molecule in the innate immune response to cytosolic DNA.^45^ In addition, enhanced immune sensing, which is induced by oxidized genomic DNA damaged by UV irradiation, is dependent on STING signaling.^24^ More recently, oxidized mtDNA was reported to participate in the STING-type I IFN axis in lupus.^26,27^ In our study, we highlight the role of the oxidized mtDNA-STING pathway in eliciting antitumor immunity in response to our irradiated tumor cell vaccine. Moreover, a recent study^49^ indicated that irradiated GM-CSF-producing cellular cancer vaccines formulated with a STING agonist cure established tumors resistant to PD-1 (programmed death ligand 1) blockade. STING signaling may represent a more attractive approach for immunotherapy or for synergy with radiotherapy^50^ or targeted therapy in clinical cancer patients.

Our study reveals a mechanism wherein oxidized mtDNA derived from irradiated tumor cells is delivered to the cytosol of DCs. In addition, oxidation of 8-OHG induced by X-ray irradiation maintains the stability of mtDNA in the cytosol of DCs. However, additional details regarding how irradiated tumor DNA is released into the cytosol of DCs and interacts with DNA sensors must be investigated further. Our study does not exclude the possibility that oxidized tumor-derived mtDNA gains access to the cytoplasm of DCs via phagocytosis when DCs engulf irradiated tumor cells. Another possible mechanism involves the CLEC9A receptor, which can mediate uptake of material from dying cells by CD8a^+^ DCs and promote cross-presentation of dead cell-associated antigens to CD8^+^ T cells.^51^ A third possibility is that tumor cell-derived DNA is transferred directly from the cytoplasm of irradiated tumor cells to the cytoplasm of DCs through gap junctions composed of connexin 43 (CX43) in a contact-dependent manner, a mechanism that is known to participate in antigen cross-presentation.^52^ Another candidate mechanism of DNA transfer involves binding to the antimicrobial peptide LL37, which leads to DNA escape from autophagy and degradation by DNase II.^53,54^ Several studies have reported that the DNA-LL37 complex activates the STING pathway.^24,53^

In summary, our findings reveal a molecular mechanism wherein oxidized mtDNA derived from irradiated tumor cells gains access to the cytosol of DCs. Oxidized mtDNA, as a DAMP or adjuvant, activates the STING-TBK1-IRF3-IFN-β pathway in DCs, which is followed by cross-presentation of irradiated tumor cell-derived antigens to CD8^+^ T cells and potentiation of the antitumor effect of the irradiated immunogenic tumor cell vaccine.

## Supplementary information

Supplemental Figures and Figure legends
