# Supplementary material for: Oxidized mitochondrial DNA sensing by STING signaling promotes the antitumor effect of an irradiated immunogenic cancer cell vaccine
Source: Cell Mol Immunol. 2020 May 12;18(9):2211–23. doi: 10.1038/s41423-020-0456-1 (PMC8429462; doi:10.1038/s41423-020-0456-1)
Supplement: Supplementary file 1 — Supplemental Figures and Figure legends [file 41423_2020_456_MOESM1_ESM.pdf]

Supplemental Figures and Figure legends

Oxidized mitochondrial DNA sensing by STING signaling promotes the antitumor effect of irradiated immunogenic cancer cells vaccine

Chunju Fang[1,2,*], Fei Mo[1,*], Li Liu[1,*], Jing Du[1], Min Luo[1], ke Men[1], Feifei Na[1], Wei Wang[1], Hanshuo Yang[1] and Xiawei Wei[1]

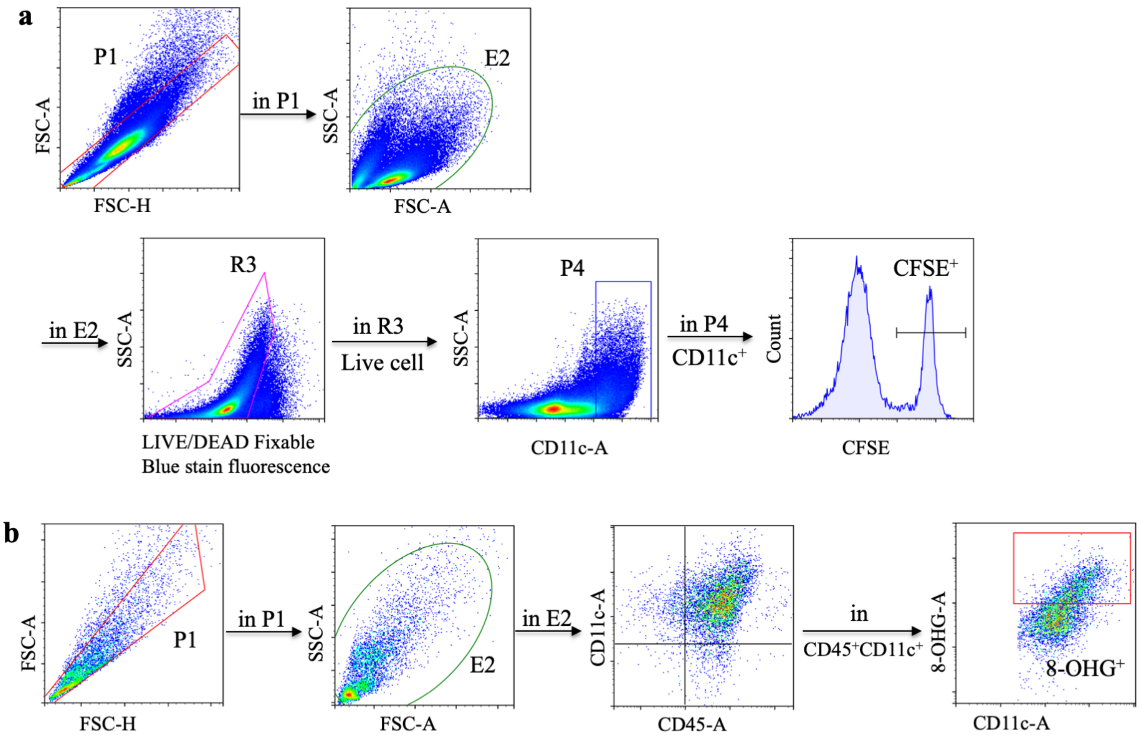

Supplementary Figure 1 Gating strategy for apoptotic cell uptake assay *in vitro* and oxidized mtDNA uptake assay *in vivo* by flow cytometry. (**a**) As showed in Figure 4a, EG7 cells were cultured with BMDCs for 3 hours *in vitro*, subsequently labeled with anti-CD11c and LIVE/DEAD™ Fixable Dead Cell Stain Kits, which excluded increased autofluorescence from dead cells after radiation. (**b**) As showed in Figure 5b, cells in the peritoneal lavage fluid of mice treated intraperitoneally with EG7 cells were labeled with anti-CD45, anti-CD11c and anti-8-OHG, and detected by flow cytometry. Gating strategy for the identification of $CD45^{+}CD11c^{+}8\text{-}OHG^{+}$ DCs are shown.

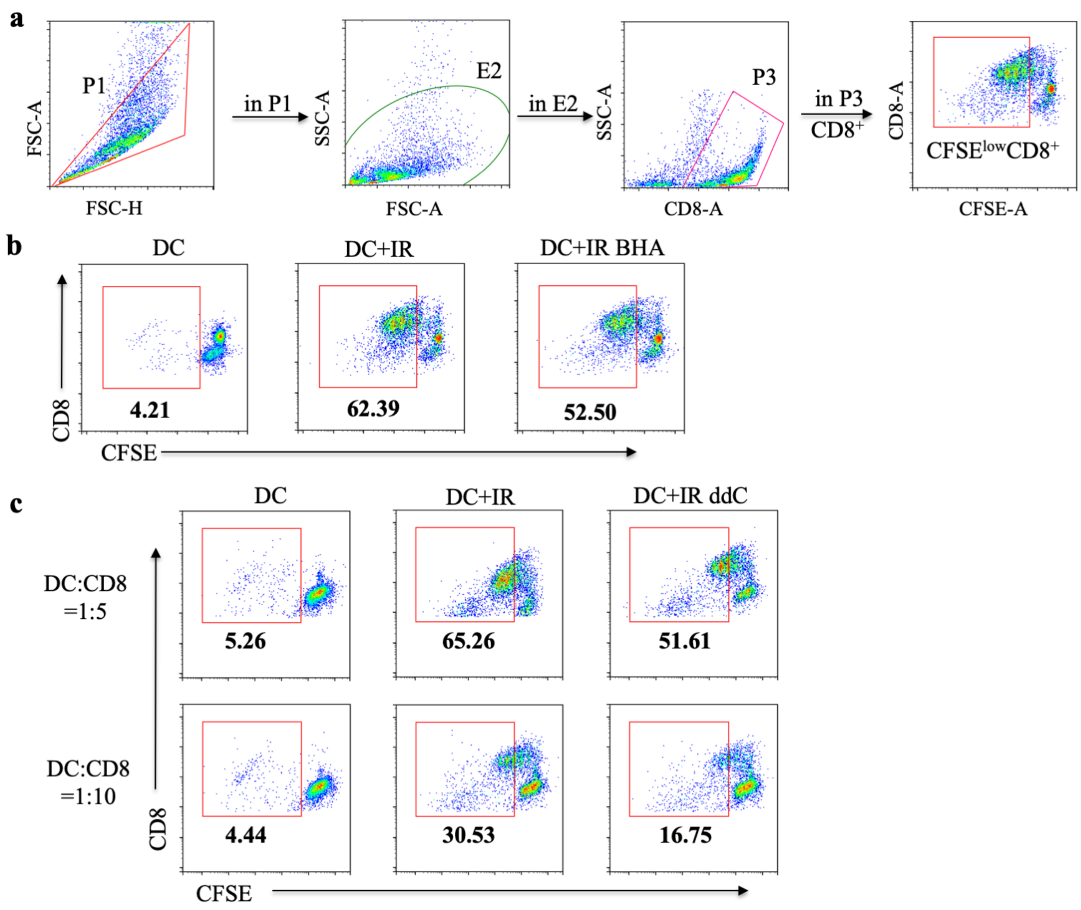

Supplementary Figure 2 Gating strategy for $CD8^+$ T cell proliferation. (**a**) As showed in Figure 6e, we estimated the proliferation of $CD8^+$ T cells by measuring the percentages of $CFSE^{low}CD8^+$ T cells in $CD8^+$ T cells by flow cytometry. Gating strategy for the identification of $CFSE^{low}CD8^+$ T cells are shown. EG7 cells treated with BHA-plus radiation (**b**) or ddC-plus radiation (**c**) were also cultured with BMDCs from WT mice (WTDCs) for 18 hours. The percentages of $CFSE^{low}CD8^+$ cells in $CD8^+$ T cells are shown.
